# Microfluidic-Based Electrochemical Immunosensing of Ferritin

**DOI:** 10.3390/bios10080091

**Published:** 2020-08-05

**Authors:** Mayank Garg, Martin Gedsted Christensen, Alexander Iles, Amit L. Sharma, Suman Singh, Nicole Pamme

**Affiliations:** 1 CSIR-Central Scientific Instruments Organisation, Sector 30-C, Chandigarh 160030, India; mayankgarg93@gmail.com (M.G.); amitsharma_csio@yahoo.co.in (A.L.S.); 2 Academy of Scientific and Innovative Research (AcSIR), Ghaziabad 201002, India; 3 Department of Chemistry and Biochemistry, University of Hull, Cottingham Road, Hull HU6 7RX, UK; martingedstedchristensen@gmail.com (M.G.C.); a.iles@hull.ac.uk (A.I.)

**Keywords:** amine functionalization, graphene oxide, immunosensor, electrochemistry, ferritin, microfluidics

## Abstract

Ferritin is a clinically important biomarker which reflects the state of iron in the body and is directly involved with anemia. Current methods available for ferritin estimation are generally not portable or they do not provide a fast response. To combat these issues, an attempt was made for lab-on-a-chip-based electrochemical detection of ferritin, developed with an integrated electrochemically active screen-printed electrode (SPE), combining nanotechnology, microfluidics, and electrochemistry. The SPE surface was modified with amine-functionalized graphene oxide to facilitate the binding of ferritin antibodies on the electrode surface. The functionalized SPE was embedded in the microfluidic flow cell with a simple magnetic clamping mechanism to allow continuous electrochemical detection of ferritin. Ferritin detection was accomplished via cyclic voltammetry with a dynamic linear range from 7.81 to 500 ng·mL^−1^ and an LOD of 0.413 ng·mL^−1^. The sensor performance was verified with spiked human serum samples. Furthermore, the sensor was validated by comparing its response with the response of the conventional ELISA method. The current method of microfluidic flow cell-based electrochemical ferritin detection demonstrated promising sensitivity and selectivity. This confirmed the plausibility of using the reported technique in point-of-care testing applications at a much faster rate than conventional techniques.

## 1. Introduction

Anemia is a key healthcare challenge in India, especially for women, and is the top cause for maternal deaths in India (~50%) [1]. Other than India, countries like Bangladesh, Nepal, Bhutan, Afghanistan, many African, and some Central American counties face severe anemia prevalence in pregnant women (WHO Global Database on Anemia, World Health Organization). Furthermore, the highest prevalence of anemia is seen in Africa followed by Southeast Asia (according to the World Health Organization). Ferritin is one of the key biomarkers for anemia and it is an iron storing and transport protein [2]. It helps in preventing iron overload and associated toxicity effects [3], and is very critical in pregnancy and thus requires regular monitoring [4]. Apart from being an active biomarker for anemia, it also indicates level of oxidative stress. According to WHO guidelines, the normal level for ferritin should be between 15 and 200 ng·mL^−1^ for males and 15 and 150 ng·mL^−1^ for females, respectively. Imbalance in its concentration is associated with a range of fatal diseases, including cardiovascular disease [5], chronic kidney disease [6], Still’s disease [7], and hemophagocytic syndrome [8]. In cancer also, ferritin is known to be upregulated and has been reported to be a marker for the diagnosis of this deadly disease [9]. Low levels of iron in pregnancy are readily treatable with tablets or diet recommendation to help fetal development. In a country like India, people often have to be assisted to attend health care facilities for check-ups. This results in loss of daily wages not only of the patient but also the person accompanying them, and thus causes a significant financial burden. The vision for the research conducted here is to develop a biosensor to be distributed by NGOs or other health monitoring agencies with access to the rural population.

Like many clinical markers, ferritin is conventionally detected using an Enzyme-Linked Immunosorbent Assay (ELISA). However, it requires not only skilled personnel but also involves use of specialized reagents such as enzyme linked antibodies and chromogens which make the method costly and time consuming [10]. The ELISA plate used for measuring an analyte has to be treated first to encourage binding of the biomolecules to the well surface. Following treatment, it then needs to be pre-coated with the primary antibodies. Once an antigen binds to the primary antibodies, secondary labeled antibodies and a chromogen are required to generate a color. Radioimmunoassays (RIA) on the other hand require usage of radiolabeled antibodies which are hazardous for daily handling. Though these conventionally used techniques are sensitive and selective to a large extent, generally they are not portable or do not provide a fast response. There is therefore a need for a dynamic, portable, and sensitive system for ferritin detection. Electrochemical methods are often attractive in clinical diagnostics settings, due to their ease of operation, minimal sample preparation, and easy data interpretation [11]. Recently, ferritin detection using electrochemical techniques has gained attraction [12,13], employing static systems [12,13,14,15,16,17,18]. Researchers of this manuscript have also recently reported the use of biosurfactant functionalized tungsten disulfide quantum dots for the electrochemical detection of ferritin using screen printed electrodes as a platform [19]. However, the availability of biosurfactant for synthesis of quantum dots is a limitation as the yield of biosurfactant is low from the microbe in use.

Continuous flow systems for in-line measurements in real-time allow running of patient samples one after another in a clinical setup. Thus, here, we investigated the electrochemical analysis of ferritin in continuous flow using a lab-on-a-chip-based approach with a microfluidic flow cell with an integrated, yet, readily exchangeable electrode. Lab-on-a-chip-based analysis platforms have been developed previously for electrochemical sensing of hydrogen ions, metals, nitrate and nitrite ions, phenolic compounds, pesticides and herbicides, and bacteria [20], including electrochemical detection of cancer biomarkers (carcinoembryonic antigen, prostate specific antigen, and cancer antigen 15-3 (CA15-3) [21]. Ko et al. reported ferritin immunosensing using a polymeric microfluidic device with gold electrodes [22]. However, the process of electrode modification and chip design are very complex. In our work, the lab-on-a-chip platform was integrated with electrochemically active carbon-coated screen-printed electrode (SPCE). The screen-printed electrodes provide a cost-effective platform for electrochemical measurements, as they are mass produced with minimum batch to batch variation, can be purchased in bulk, and can be read out with portable potentiostats, enabling point-of-care or even in-the-field application [23,24]. Owing to these advantages, researchers have combined screen-printed electrodes with flow cells for the electrochemical detection of various analytes [25,26,27,28,29]. Most of these flow cells allow screen-printed electrodes to be integrated in such a fashion that the flow cell cannot be reused once the measurements are made. This means that both the electrode and flow cell have to be disposed of. Furthermore, the flow cell designs are very intricate and do not provide a very controlled flow over the electrode surface. Thus, there is a need for a simple flow cell with integrated screen-printed electrodes for analysis of biomarkers such as ferritin, which allows ready exchange of electrodes and also provide a controlled flow over the electrode surface to enable precise control of time for binding and washing steps. 

Therefore, we employed a simple flow cell and a magnetic clamping mechanism, previously developed in our group for glucose analysis [30] to study the electrochemical detection of ferritin. The different concentrations of the ferritin antigen were run one after the other on the electrode system with buffer washings in between them as is required for a clinical device. To the best of our knowledge, the use of a graphene-based 2D layered material as a platform for the continuous electrochemical sensing of ferritin in a simple flow cell has not yet been reported. Here, layered materials refer to those structures wherein layers of atoms are stacked on top of one another. Based on the available literature for the continuous electrochemical detection of ferritin, it can be concluded that a simple flow cell is required which can house the functionalized electrodes, thus increasing its flexibility of use. The study therefore aims to design and fabricate a flow cell with an integrated commercially available electrode which is modified with amine-functionalized graphene oxide, onto which anti-ferritin antibodies were immobilized for the continuous flow-based detection of ferritin, which is a novelty. 

## 2. Experimental Section

### 2.1. Materials

Graphite powder was purchased from Alfa Aeser (Mumbai, India). Potassium permanganate, amino-terephthalic acid (NH_2_-BDC), and sodium nitrate were sourced from Sigma-Aldrich (Bengaluru, India). Sulfuric acid and hydrogen peroxide were obtained from Merck (Mumbai, India). Ferritin antigen, anti-ferritin antibody, myoglobin, hemoglobin, bovine serum albumin (BSA), human serum (USA origin, sterile-filtered), potassium ferrocyanide, potassium ferricyanide, ethylene diamine tetra acetic acid (EDTA), and phosphate buffer saline tablets (PBS) (pH 7.4) were bought from Sigma-Aldrich (Dorset, UK). A ferritin ELISA kit was used for the validation studies from Orgentec (Mainz, Germany). DropSens carbon-coated screen-printed electrodes (SPCE, DS110) were procured from Metrohm (Runcorn, UK). A Milli-Q reverse osmosis system was used to obtain deionized water and was used for performing all experiments (Millipore, UK). Details of NH_2_-GO synthesis and its characterization are given in Supplementary Information ESI1. 

### 2.2. Fabrication of Microfluidics Flow Cell and Surface Modification of Electrode 

**Figure 1:**
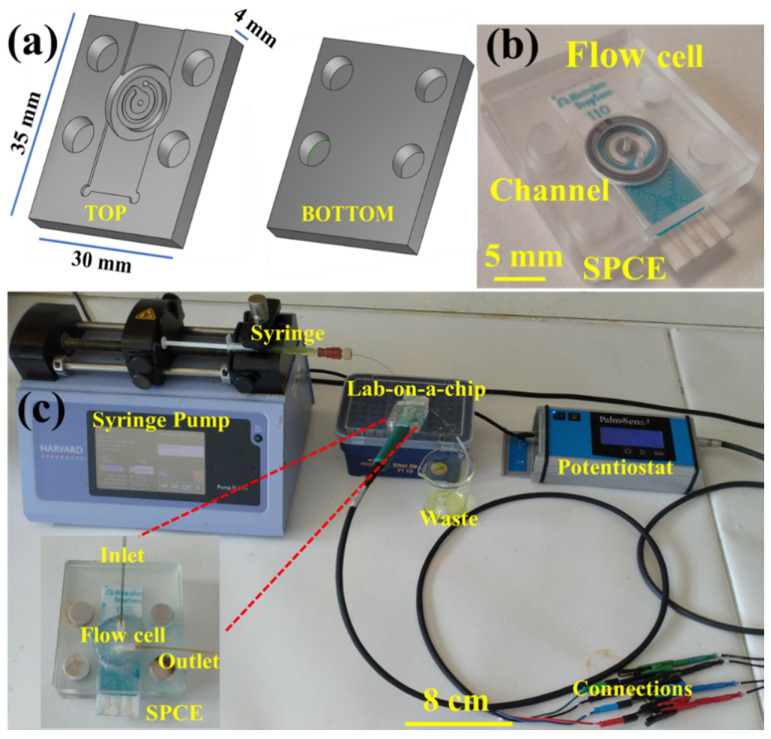
(**a**, **b**) Details of the microfluidic flow cell design. The top poly(methyl methacrylate) (PMMA) plate featured a groove to house the carbon-coated screen-printed electrode (SPCE), a spiral channel to move liquid over the working electrode surface with inlet an outlet holes, as well as four circular grooves to house the NdFeB magnets for clamping. The bottom plate was flat apart from the four magnet holding recesses. (**c**) Photograph of the experimental setup showing the syringe pump and collection vessel for fluid movement through the microfluidic flow cell with integrated SPCE electrode connected to a small potentiostat for recording of electrochemical measurements.

The flow cell was designed on AutoDesk Inventor 2019 (San Rafael, CA, USA) and was fabricated from two layers of poly(methyl methacrylate) (PMMA) via a CNC milling (Datron M7, Datron, Germany) with 0.5, 1, 3, and 6 mm carbide drill bits (Figure 1). The outer dimensions of the flow cell were 35 mm × 30 mm × 4 mm. The top layer featured a 29 mm long, 10 mm wide, and 1 mm deep groove to house the DropSens SPE electrode. This layer also featured a spiral channel for transporting liquid over the surface of the working electrode. The spiral had a 3.8 mm diameter and the channel was 0.6 mm wide and 140 μm deep, with a total length of 19.45 mm, capable of handling an internal volume of 3.30 μL. At either end, there was an inlet and outlet hole (1 mm diameter). Both plates also featured wells of 6.2 mm diameter and 3.5 mm depth to hold NdFeB magnets (6 mm diameter, 3 mm height). The NdFeB magnets were obtained from Magnet Sales (Wiltshire, UK) and were used for clamping the plates together with the electrode. An O-ring (Size BS012, Nitrile, Simrit Service Centre Cramlington Ltd., UK) was used to ensure leak-free operation. Tubing (0.3 mm i.d., 1.58 mm o.d., Supelco) and capillary (100 μm i.d. and 363 μm o.d., Polymicro Technologies) were glued into the inlet and outlet holes with Araldite glue. At the inlet site, the tubing was interfaced to a syringe (1 mL), using an adapter (Luer (Female) to 1/4”-28 flat bottom (Female)) and flangeless fitting (1/4”-28 flat bottom). At the outlet site, tubing was inserted into a collection vessel. The liquid was pumped through the microfluidic chip via a syringe pump (11 Elite, Harvard Apparatus, MA, USA) (Figure 1c). A flow rate of 10 µL·min^−1^ was applied unless otherwise stated, equating a flow speed of 1.984 mm·s^−1^ in the spiral channel. Before insertion into the chip device, the surface of a carbon coated screen-printed electrode (SPCE) with a working area of 11.34 mm^2^ was modified with the amine-functionalized graphene oxide (NH_2_-GO) by drop casting. Ten microliters of NH_2_-GO suspension prepared in deionized water (1 mg·mL^−1^) was sonicated for 30 min prior to pipetting onto the working area of the electrode, and left to air dry for ~30 min [31]. The electrodes were clamped between the PMMA plates prior to use. Electrochemical measurements via cyclic voltammetry (CV) were recorded on a PalmSens3 potentiostat (Netherlands), interfaced with a computer using PSTrace software. The screen-printed electrode was coupled to the potentiostat via DRP-CAC (Metrohm, Runcorn, UK) cable connectors.

### 2.3. Electrochemical Measurements

Cyclic voltammetry (CV) was used for electrochemical characterization of the electrodes along with ferritin sensing. Ferritin antibodies (Fer_Ab_) were immobilized on NH_2_-GO modified SPCE for ferritin (Fer) sensing. For immobilization of the ferritin antibodies, 10 µL of antibody solution (10 µg·mL^−1^) prepared in phosphate buffer was drop-casted on the NH_2_-GO modified SPCE and left for air dry for about 30 min. It is expected that the amine groups present on the NH_2_-GO will bind to the carboxyl groups present in the Fc region of the antibody, therefore creating a strong binding force for the antibody to remain on the electrode surface. For CV measurements, the phosphate buffer consisting of 1.0 mM Fe(CN)_6_^3−^/Fe(CN)_6_^4−^ as redox marker was used as an electrolyte and voltammograms were run in the potential window of −0.5 V to 0.7 V. The electrochemical characterization of the electrodes involved measuring the electrochemical response of the SPCEs after each modification; bare SPCE, NH_2_-GO@SPCE, Fer_Ab_/NH_2_-GO@SPCE, and Fer/Fer_Ab_/NH_2_-GO@SPCE. This was performed to ensure electrochemical activity of the sequentially modified electrodes, which is a prerequisite for any electrochemical sensing. For electrochemical characterization, the effect of scan rate (0.02 V·s^−1^ to 0.10 V·s^−1^) and flow rate (0.0 and 40 µL·min^−1^) on the response of the developed lab-on-a-chip embedded SPCE was also studied. The stability of the NH_2_-GO-modified electrode was verified by recording its cyclic voltammograms for 96 cycles from −0.5 V to 0.5 V at a scan rate of 0.04 V·s^−1^.

For ferritin quantification, different concentrations of ferritin were prepared in a redox probe containing buffer. A redox probe containing buffer without any ferritin was used as a blank. Solutions were loaded into the syringe and pumped through the microfluidic device at 10 µL·min^−1^. This flow rate had been optimized and is discussed below. In between measurements, the electrode surface was washed with phosphate buffer for 10 min. The effect of buffer pH (pH 5.5 to pH 8.5) and interfering agents (hemoglobin, BSA, and myoglobin) on the CV signal were also studied. For this, the pH of the redox probe containing buffer was adjusted with hydrochloric acid and sodium hydroxide. The interferents were added to the sample solutions at a concentration of 500 ng·mL^−1^.

To check the reusability of the sensor, regeneration studies were performed. For this, after pumping a certain concentration of ferritin over the electrode and recording its response, a solution of 30 mM EDTA was introduced to remove the bound antigen. Then, PBS was pumped through the device for washing, followed by pumping of ferritin analyte solution. This cycle was repeated five times. The sensor performance was further validated by comparing the results with those obtained from a commercial ELISA kit. For serum studies, human serum samples were aliquoted and kept in the freezer until use and were thawed prior to use. A known concentration of ferritin was spiked into the serum to obtain samples with different ferritin concentration and the current response was recorded as before. All the experiments were performed at room temperature (~20 °C). 

### 2.4. Computer Simulations

Computer simulations were carried out in COMSOL Multiphysics 5.2 (COMSOL AB). Mesh convergence assessments were performed on simulation geometries to determine the appropriate mesh settings in each of the simulations. COMSOL’s physics for laminar flow and transport of diluted chemical species were employed for the simulations. The computed flows were calculated based on the assumption of a non-compressible fluid and with a no-slip boundary condition imposed on the wall domains. The transport of ferritin in the simulation was described by the generic diffusion equation (Equation (1)) with R as a reaction term, $\vec{u}$ as the velocity field, and *c* is the concentration of the species.

(1) $$\frac{dc}{dt}=\nabla \cdot (D\nabla c)-\nabla \cdot (\vec{u}c)+R$$

## 3. Results and Discussion

### 3.1. Characterization of Amine-Functionalized Graphene Oxide (NH_2_-GO)

The amine-functionalized graphene oxide was characterized via spectroscopy, X-ray diffraction analysis, and electron microcopy as laid out in the experimental section. The detailed results of UV/Vis spectra (Figure S1), FTIR spectra (Figure S2), Raman spectra (Figure S3), XRD pattern (Figure S4), and TEM images (Figure S5) are discussed in the Supplementary Information.

### 3.2. Computer Simulations

**Figure 2:**
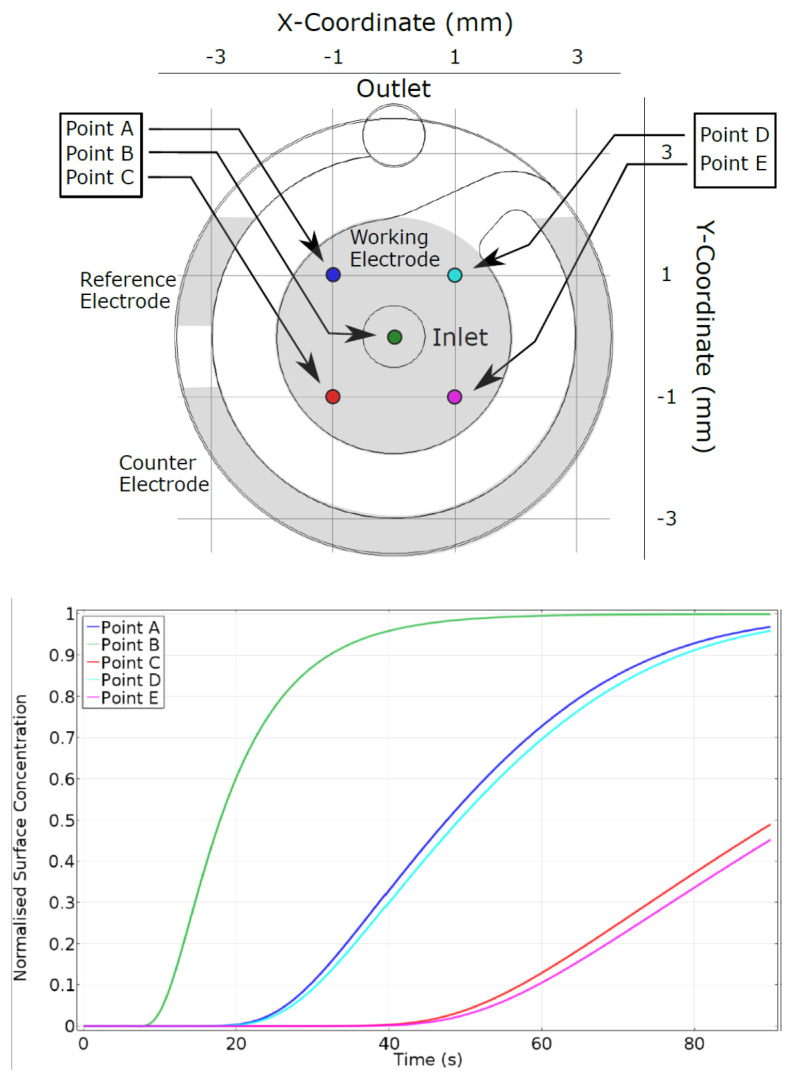
COMSOL simulations showing (**a**) the simulation geometry with five simulation probe points. The location of the reference, working and counter electrode at the bottom of the flow cell are shown in grey. (**b**) Evolution of concentration over time (normalized) at the five simulation points.

COMSOL was used to study the flow profile and changes in the concentration of the liquid inside the flow cell. For the simulations, the probe points were set at the inlet port as well as spread across the spiral channel in the flow cell (Figure 2). The simulation showed the evolution over time of the concentration at five distinct points on the electrode surface. The results demonstrate that a concentration of 50% is attained at the center surface point following approximately 17 s with 100% being achieved at 60 s. In comparison, the two peripheral surface points closest to the outlet attained 50% concentration at ~47 s, whereas the two furthest away peripheral surface points took more than 90 s.

Furthermore, it is worth noting from the modeling that the spiral channel, intended to effectively guide the flow over the counter and reference electrode, did not fully achieve this aim. This is likely to be due to the gap of ~500 µm in our design between the top and bottom plates of the magnetically clamped assembly, caused by the O-ring seal. The spiral channel is thus located above a 500 µm high circular chamber above the electrode. The design and thus guidance of the flow could be improved by the application of a thinner O-ring, a stronger clamping force via stronger magnets or a change to nuts and bolts to reduce the height of this gap. This would also reduce diffusion distances and diffusion times significantly and increase the sensitivity and speed of the sensor response.

### 3.3. Electrochemical Characterization 

#### Effect of Scan Rate

The effect of scan rate on electrode response was studied for a bare SPCE, as well as for SPCEs functionalized with NH_2_-GO@SPCE and Fer_Ab_/NH_2_-GO@SPCE. The scan rate was varied from 0.02 V·s^−1^ to 0.10 V·s^−1^. The obtained cyclic voltammograms, as well as plots of current versus scan rate and current versus square root of scan rate, are shown in Figure S6. The cyclic voltammograms showed oxidation and reduction peaks at 0.3 V·s^−1^ and −0.1 V·s^−1^, respectively, corresponding to the conversion of Fe^3+^ to Fe^2+^ and vice versa (Figure S6a,d,g). With increased scan rate, the current increased in all the electrodes (Figure S6b,e,h), while the redox potential changed in position. The increase in current signal with increase in scan rate, can be accounted for decrease in diffusion layer at the electrode surface, as a result of which more current flows [32,33]. The R^2^ value for the plots of current versus square root of scan rate was observed to be higher as compared to current versus scan rate, as expected by the Randles–Sevcik equation, which states that for reversible electrochemical reactions, the current increases linearly with the square root of the scan rate. This can be extended to the state that our system has free diffusing species and the electroactive species are not limited to the electrode surface [32,33].

#### Effect of Flow Rate

The effect of flow rate through the channels on the electrode signal was studied next. A phosphate buffer containing redox probe was pumped at different flow rates (0.0 and 40 µL·min^−1^) through the device with a non-functionalized SPCE. The I_pa_/I_pc_ ratios were calculated and the observed trend is shown in Figure S7. The ratio of I_pa_/I_pc_ is linked to the reversibility of the reactions taking place at the electrode surface; a ratio close to 1 is expected for a highly reversible process. It is clearly evident from Figure S7 that at flow rate of 10 µL·min^−1^, the ratio for the anodic to the cathodic current is closest to 1. Thus, this flow rate was chosen for further experimentation purposes.

#### Stability of NH_2_-GO Coating

**Figure 3:**
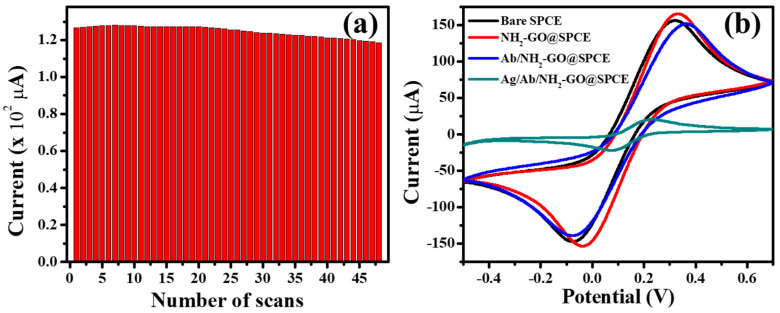
(**a**) Effect of number of scans on stability of NH_2_-GO-modified SPCE. (**b**) Cyclic Voltammogram (CV) response with varying flow rates and Cyclic Voltammogram of sequentially modified electrode (Bare SPCE, NH_2_-GO@SPCE, Fer_Ab/_NH_2_-GO@SPCE, and Fer/Fer_Ab_/NH_2_-GO@SPCE, respectively) in buffer containing redox marker at a scan rate of 0.04 V·s^−1^.

The stability of the NH_2_-GO coating on the electrode was checked by continuously pumping the redox marker over the modified electrode and 48 scans were run. For up to 25 scans, the response was constant (Figure 3a); thereafter, a slight decrease in electrochemical response started to appear. On reaching the 48^th^ scan, a 6.7% decline in current was observed. This suggests that the NH_2_-GO coating on the electrode is sturdy and stable, and thus potentially suitable as a biosensor for continuous detection of an analyte over an extended period of time. The stability of the Fer_Ab_/NH_2_-GO was not evaluated. This can be attributed to fact that the amine groups present on the NH_2_-GO were able to covalently bind to the carboxyl groups on the antibody. This ensured a strong binding force and hence the check on stability of the surface after antibody immobilization was not considered. This is in line with the work done by Gupta et al. wherein they used NH_2_-GO as a platform for the direct immobilization of anti-*E. coli* antibodies [34].

#### Effect of Functionalization and Antibody Immobilization

To study the effect of functionalization and antibody immobilization, cyclic voltammograms were recorded for all the sequentially modified electrodes, i.e., bare SPCE, NH_2_-GO@SPCE, Fer_Ab_/NH_2_-GO@SPCE and Fer/Fer_Ab_/NH_2_-GO@SPCE (Figure 3b). The electrochemical response of bare SPCE increased when modified with NH_2_-GO, which can be explained by the role of lone pair of electrons present on the nitrogen atom. This easily available lone pair of electrons participates in the electron transfer processes taking place at the electrode surface resulting in an increase in the conduction of the system. The introduction of ferritin antibodies on the electrode however resulted in a decrease in current, which is attributed to the insulating effect caused by biological molecules [35]. Though we could not calculate the antibody density on the electrode surface, work done by Chouda et al. reports an antibody density of 4.8 × 10^12^ Ab/cm^2^ for an immunosensor for *Staphylococcus aureus*. The authors used charge transfer resistance values before and after antibody immobilization to calculate the antibody coverage on the electrode [36]. On the addition of antigen, an immunocomplex forms between antigen and antibody, which reduces the current significantly, further hindering the flow of current [37].

#### Electrochemical Detection of Ferritin and Sensor Performance

**Figure 4:**
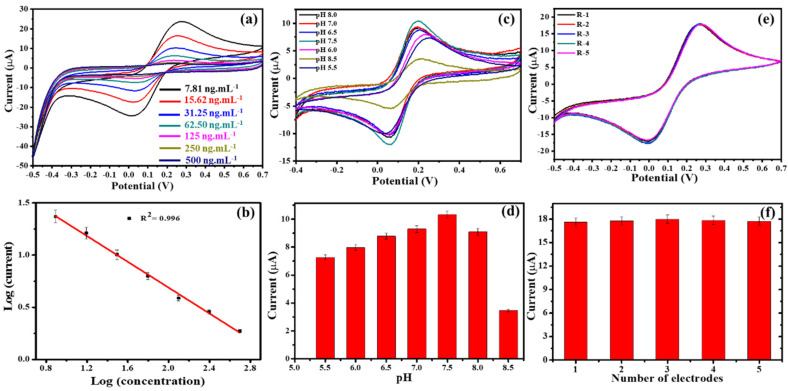
(**a**) Cyclic voltammograms from Fer/Fer_Ab_/NH_2_-GO@SPCE in presence of ferritin (7.81–500 ng·mL^−1^). (**b**) Logarithmic plot of ferritin concentration versus obtained current. (**c**) Cyclic voltammograms and (**d**) bar chart of obtained currents when varying the pH between pH 5.5 and pH 8.5. (**e**) Cyclic voltammograms and (**f**) bar charts of currents obtained from five separately prepared electrodes to confirm reproducibility.

The microfluidic flow cell was next tested for quantitative electrochemical analysis of ferritin. The Fer/Fer_Ab_/NH_2_-GO@SPCE was fitted into the flow cell and different concentrations of ferritin prepared in phosphate buffer consisting of 1.0 mM Fe(CN)_6_^3−^/Fe(CN)_6_^4−^ were pumped at 10 µL min^−1^ over the electrode whilst the cyclic voltammograms were recorded (Figure 4a). The current decreased as the concentration of ferritin was increased (Figure 4b) as expected from the insulating effect of the immune complex [35]. The linearity range obtained was 7.81 to 500 ng·mL^−1^, which covers the clinically relevant range. An R^2^ value of 0.996 was obtained with the limit of detection (LOD) of 0.413 ng·mL^−1^. The LOD, a value which indicates the lowest quantity of an analyte, differentiable from the absence of that substance, was calculated using the formula: LOD = 3.3 × (Standard deviation/slope). As per the IUPAC nomenclature, the LOD is defined as the minimal detectable value as the mean blank value plus three times the standard deviation [38,39,40,41].

Further, the effect of pH on the response of the lab-on-a-chip-based immunosensor was studied (Figure 4c,d). As the pH was changed from acidic (pH 5.5) to neutral (pH 7.5), the current response increased, with the maximum response observed at pH 7.5. When moving towards the alkaline pH range, i.e., pH 8.0 and pH 8.5, the current signal decreased, which could be attributed to denaturation and instability of the proteins at non-physiological conditions [35]. Ferritin has a pI of ~5.5, thus the increasing negative charge at the higher pH values will increase electrostatic repulsion between the participating moieties and hence the current decreases [42]. The same trend with pH variation was also observed by Garg et al. [19]. The sensor reproducibility was verified by preparing five electrodes in the same way and recording their response (Figure 4e,f). It was found that all the electrodes gave a similar response with a relative standard deviation of 0.74%.

**Figure 5:**
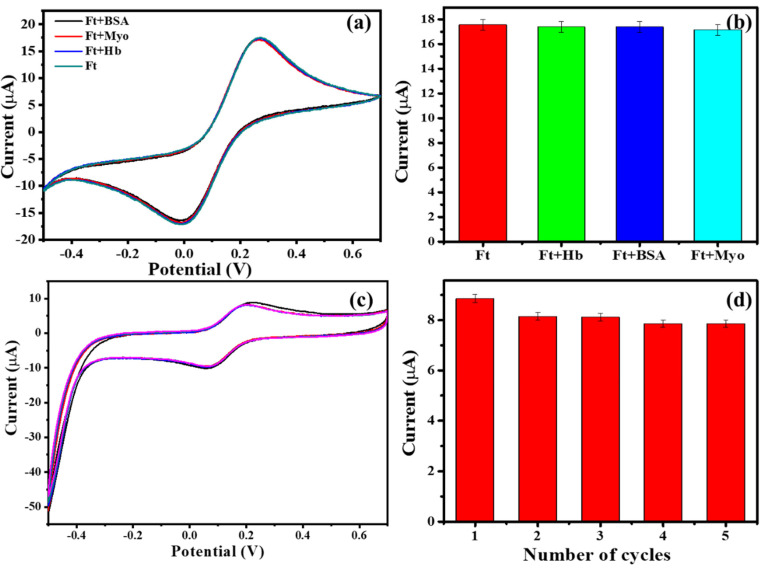
(**a**) Cyclic voltammograms and (**b**) bar chart of currents of obtained from ferritin in the absence and presence of interferents, i.e., hemoglobin (Hb), myoglobin (Myo), and bovine serum albumin (BSA). (**c**) Cyclic voltammograms and (**d**) bar chart of currents of obtained five cycles of ferritin analysis and regeneration.

The selectivity of the immunosensor chip device towards ferritin was confirmed by interference studies with oxygen binding proteins such hemoglobin, myoglobin, and bovine serum albumin (Figure 5a,b). It was observed that these interferents had a negligible effect on the sensor readout. The reusability of the sensor was evaluated using regeneration following EDTA and PBS wash, as described in the experimental section. As can be seen from Figure 5c,d, after five cycles of regeneration, the current response dropped to ~11% as compared to the initial point. This hints at the possibility of reusing the sensor device for a number of scans before a change of electrode would be required.

The practical applicability of the biosensor device was investigated with ferritin spiked into human serum at different concentrations, i.e., 31.25 ng·mL^−1^, 62.50 ng·mL^−1^, and 125 ng·mL^−1^. The obtained signal was converted to a quantitative readout via the calibration curve as shown in Figure 4b. The current obtained from these spiked serum samples was used to calculate the concentration of ferritin in those samples by using the linear equation obtained from the standard calibration curve. This resulted in a deviation of ~5–10% with potential for further optimization (Table 1).

For further evaluation, sensor response was compared to that of a commercially available ELISA kit. The linear range in case of the ELISA was 0 to 1500 ng·mL^−1^ with an R^2^ value of 0.966 (Figure S8). Though the linear range was higher than the present developed method, the major disadvantage for the commercially available ELISA method is the use of secondary conjugated antibodies and a chromogenic substrate to generate a readable signal. Moreover, for microplate readings used in ELISA tests, a microplate reader is required, increasing the cost of the overall method. The lab-on-chip device reported in the present manuscript on the other hand, uses only primary antibodies specific to the ferritin antigen for the bio-recognition reactions. The handheld potentiostat employed with lab-on-chip is not only portable but is also economical.

The current immunosensor performance is comparable with the previously reported microfluidic or flow-based electrochemical sensors for ferritin. Ko et al. designed and fabricated a PDMS/PMMA-laminated microfluidic device having an integrated gold-based electrode in the chip. The electrode was immobilized with ferritin antigen and two sets of antibodies, anti-ferritin IgG and anti-IgG antibody labeled with HRP, were used. The system relied on the precipitation reaction to generate the signal. The work however suffered from the drawback of non-reusability of the electrode, which hinders its usage as a point-of-care device [22]. The work by Reymond et al. on a microfluidic-based ferritin immunoassay used an eight channel electrochemical microchip modified with magnetic and non-magnetic microspheres for antibody immobilization. However, the research did not cover the required clinical range of ferritin quantification [43]. In another article by Song et al., the use of cotton thread as a microfluidic circuit was suggested, where three sets of antibodies were used in the sensor fabrication. Though the linear range achieved by this sensor is very high (5 to 5000 ng·mL^−1^), the use of multiple antibodies and a complex system increases the overall cost of the system [12]. The most recent report for the detection of ferritin in a microfluidic fashion is the use of a paper-based electrode system. The electrode was functionalized with graphene and further modified with EDC/NHS for antibody immobilization to take place. This sensor performance was better than in our study in terms of both linear range as well as the obtained LOD value. The main disadvantages of this work are the multiple electrode preparation steps and the inability to change the electrode in the same microfluidic device [44].

Based on the literature available for the microfluidic-based electrochemical detection of ferritin, we can state that most of these were based on multiple steps for electrode functionalization and employed different chemistries for antibody immobilization. Moreover, the systems lacked easy replacement of electrodes. The immunosensor reported in the present manuscript is advantageous over other reported immunosensors for ferritin in terms of covering the clinical ferritin range, and one step process for both functionalization and immobilization. The amine functionalized graphene oxide plays a dual role of electrode surface modifier and as well as immobilization matrix. The major highlight of the current method is the continuous electrochemical detection of ferritin as compared to the static-based systems mentioned in the introduction section. The current system has the microfluidic channels manufactured into the top chip which allows easy electrode swapping, thereby giving flexibility in terms of usage. We were able to demonstrate a dynamic method for the sensing of ferritin which can be further improved upon, to develop a system for clinical settings. This device can be useful in a small clinical setting with a lightly trained skilled technician. This system with further improvements in the design (to make portable) can be used as a point-of-care technology. Moreover, the system is open ended and can also be used for sensing of other analytes by changing the bioreceptor.

## 4. Conclusions

An electrochemical microfluidic device for the sensitive determination of ferritin is demonstrated. The microfluidic device enabled ferritin estimation in continuous flow, which will be helpful for continuous monitoring. Furthermore, the amine functionalization of SPCE, integrated with the lab-on-chip device, provided a platform for both electrode surface modification and immuno-sensing to take place, thus simplifying the overall analysis process. The lab-on-chip with integrated NH_2_-GO-functionalized SPCE is capable of sensing the ferritin in the range 7.81 to 500 ng·mL^−1^, which covers the clinically relevant range and is found to be robust against pH changes and interferents. Validation of the device was performed by comparing its response with the results from a standard ELISA kit. The sensor response with spiked human serum samples showed promising results for consideration for use in real time applications that can be operated even by nurses in small/local healthcare facilities without requiring sophisticated machinery. This is a better alternative as compared to a central facility where samples would have to be transported. Moreover, the device is open ended in terms of applicability as simply by changing the bio-receptor, it can also be used for other biomarkers.

## Figures and Tables

**Table 1:** Results for ferritin spiked into human serum samples.

Concentration Added (ng·mL^−1^)	Concentration Found (ng·mL^−1^)	% Found
31.25	34.47	110.30
62.50	70.91	113.45
125	120.27	96.21

## Supplementary Materials

The following are available online at https://www.mdpi.com/2079-6374/10/8/91/s1, Figure S1: UV/vis absorption of graphene oxide (GO), NH_2_-BDC and amine functionalized graphene oxide (NH_2_-GO), Figure S2: FTIR spectra of graphene oxide (GO), NH_2_-BDC, and amine functionalized graphene oxide (NH_2_-GO), Figure S3: Raman spectroscopy of graphene oxide (GO) and amine functionalized graphene oxide (NH_2_-GO), Figure S4: XRD analysis of graphene oxide (GO), amine BDC (NH_2_-BDC), and amine functionalized graphene oxide (NH_2_-GO), Figure S5: TEM images of NH_2_-GO, Figure S6: Effect of scan rate studies, Figure S7: Effect of flow rate, Figure S8: Calibration curve from ELISA kit
