# Supplementary material for: Microfluidic-Based Electrochemical Immunosensing of Ferritin
Source: Biosensors (Basel). 2020 Aug 5;10(8):91. doi: 10.3390/bios10080091 (PMC7460419; doi:10.3390/bios10080091)
Supplement: Supplementary file 1 [file biosensors-10-00091-s001.pdf]

Supplementary Information:
Microfluidic-based Electrochemical Immunosensing of Ferritin

Mayank Garg[1,2,3], Martin Gedsted Christensen[3], Alexander Iles[3], Amit L. Sharma[1,2], Suman Singh[1,2*], Nicole Pamme[3*]

[1]CSIR- Central Scientific Instruments Organisation, Sector 30-C, Chandigarh-160030, India

[2]Academy of Scientific and Innovative Research (AcSIR), Ghaziabad-201002, India

[3] Department of Chemistry and Biochemistry, University of Hull, Cottingham Road, Hull, HU6 7RX, U.K.

*Corresponding authors: n.pamme@hull.ac.uk, ssingh@csio.res.in

1. Synthesis and Characterization of Amine-Functionalized Graphene Oxide (NH_2-GO)

The graphite was first converted to graphene oxide (GO) using a modification in Hummer's method [1]. For the synthesis of amine functionalized graphene oxide, an adapted version of the previous method was used [2,3] to explore the use of amino-terephthalic acid as a potential source for amine groups. A dispersion of graphene oxide prepared in deionized water (1 mg·mL^{-1}) was subjected to probe sonication followed by addition of amino-terephthalic acid prepared in deionized water (1 mg·mL^{-1}). This mixture was stirred at 60 °C for 16 h. After stirring, the solution was centrifuged at 7000 rpm for 15 min and washed several times with water via sonication, followed by air drying. This material is referred as NH_2-GO in the manuscript.

For functional group verification, the synthesized NH_2-GO was characterized via UV/vis (U-3900H, Hitachi, Japan) in the 200-500 nm range, FT-IR (Perkin Elmer Spectrum II, Massachusetts, USA) in the 4000-400 cm^{-1} wavenumber range and Raman spectroscopy (Spectral Analyzer, Renishaw, Wotton-under-Edge, UK) in the 1300-1600 cm^{-1} range. For UV/vis spectroscopy, 1 mg·mL^{-1} of GO, NH_2-BDC and NH_2-GO were prepared in Milli-Q water and spectra was recorded with Milli-Q water for baseline correction. For FT-IR measurements, 1 mg of GO, NH_2-BDC and NH_2-GO were pelleted with IR grade potassium bromide. For Raman measurements, the samples were placed onto the glass slide and their Raman signal was recorded. The crystal structures of GO, NH_2-BDC and NH_2-GO were studied via XRD (Bruker D8 Advance, Germany) over the 5° to 50° 2θ range. TEM images (JEOL, JEM 2010, Japan) were recorded to understand the morphology of the synthesized material. For sample preparation, NH_2-GO was dispersed in Milli-Q water and sonicated for 30 min prior to drop casting onto a lacy carbon TEM grid and left for air dry.

The amine functionalized graphene oxide material was analyzed via UV/vis absorbance (Figure S1), FTIR (Figure S2), Raman (Figure S3), XRD (figure S4) and TEM (figure S5).

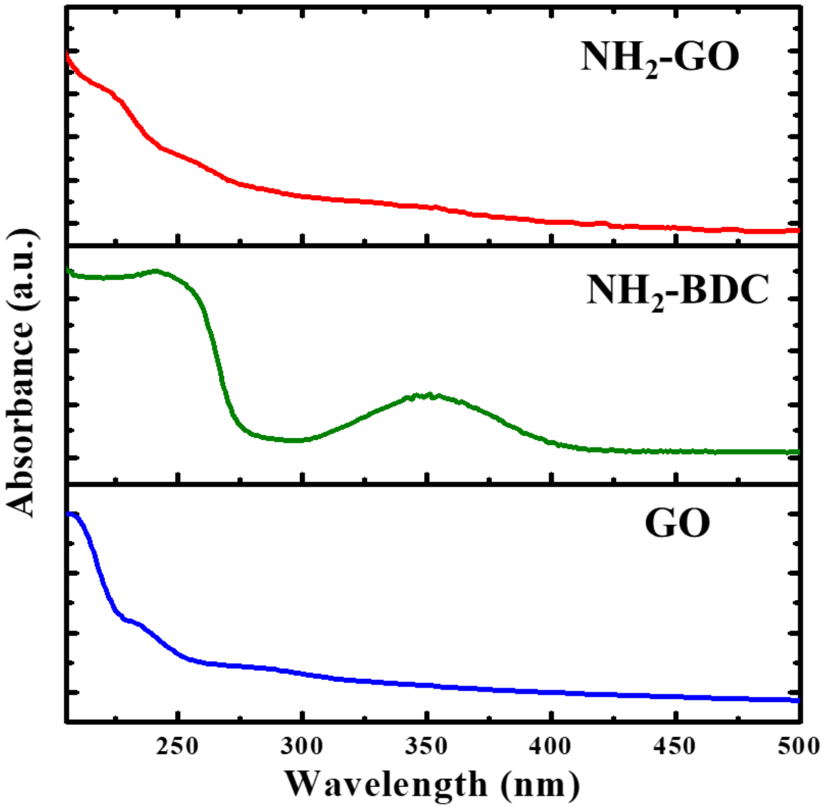

Figure S1. UV/vis absorption of graphene oxide (GO), NH_2-BDC and amine functionalized graphene oxide (NH_2-GO), showing the absorbance peaks of individual material. In case of GO, the π- π* transitions occurring in aromatic C–C bonds gives peak at around 230 nm whereas a faint shoulder at around 300 nm is characteristic of the n- π * transitions of C═O bonds [4]. NH_2-BDC gives peak at around 350 nm which matches well with previous report [5]. In case of NH_2-GO, it displays a UV/vis spectrum which encompasses peaks both from GO and NH_2-BDC showing the successful synthesis and functionalization of the material.

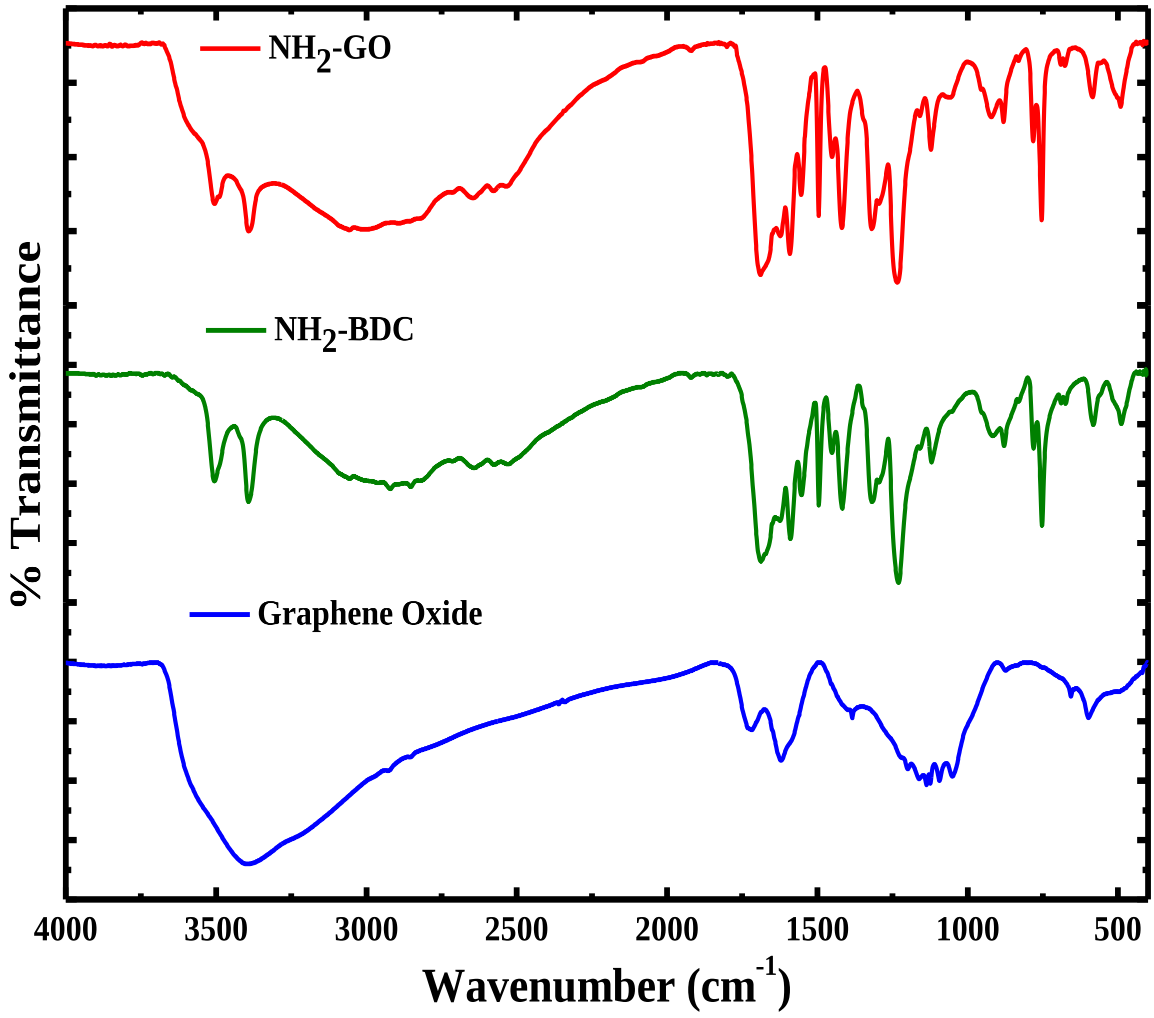

Figure S2. FTIR spectra of graphene oxide (GO), NH_2-BDC, and amine functionalized graphene oxide (NH_2-GO), showing functional groups present in these samples. Graphene oxide has functional groups such as O-H, C=C and C=O at around 3402, 1624 and 1726 cm^{-1}, respectively [6]. NH_2-BDC contributes additional functional groups such as N-H and C-N which gives peaks at 3385 and 1226 cm^{-1} [5]. The NH_2-GO has N-H, C=O, O-H and C-N functional groups at 3385, 1692, 3504 and 1226 cm^{-1}, respectively [7]. All these functional groups are essential for facilitating the binding of the antibody on the material. It is clearly evident that the amino-terephthalic acid was able to functionalize the graphene oxide.

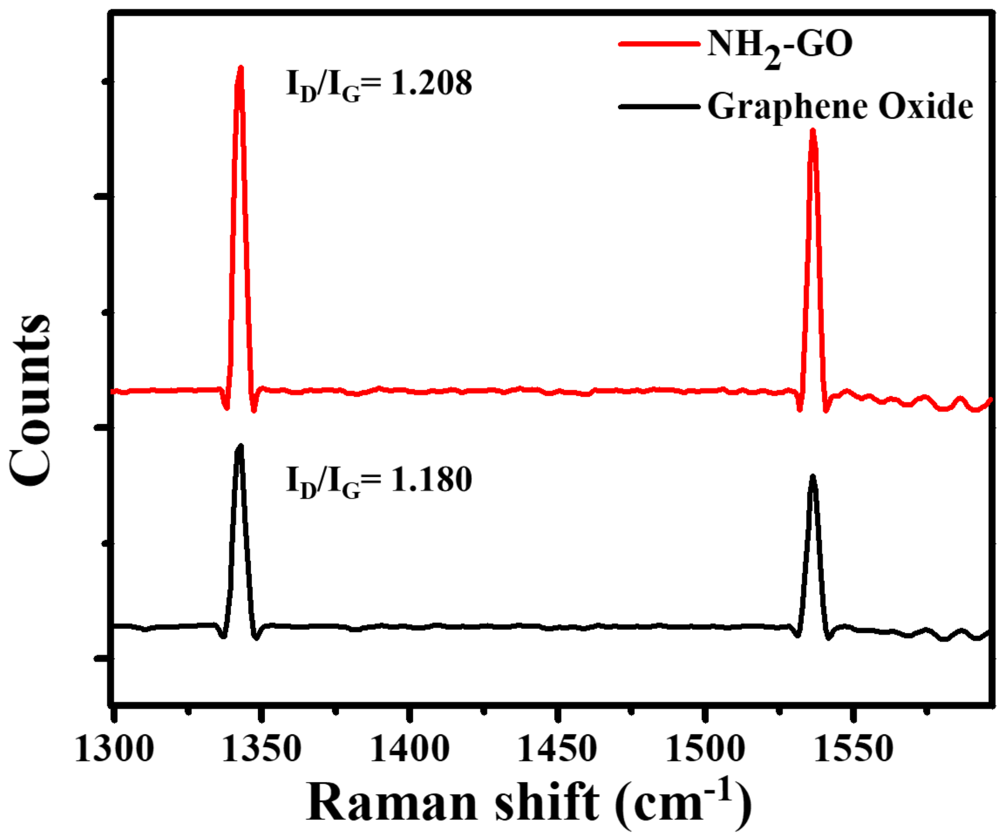

Figure S3. Raman spectroscopy of graphene oxide (GO) and amine functionalized graphene oxide (NH_2-GO), showing characteristic D (defects) and G (graphene) bands at 1342 and 1536 cm^{-1}, respectively [8]. The I_D/I_G ratio for GO was calculated to be 1.180, which increased to 1.208 after its amine functionalization. This might be due to the introduction of nitrogen in the graphene structure, thus leading to generation of more defects. With increase in defects, the signal of D band increases [9].

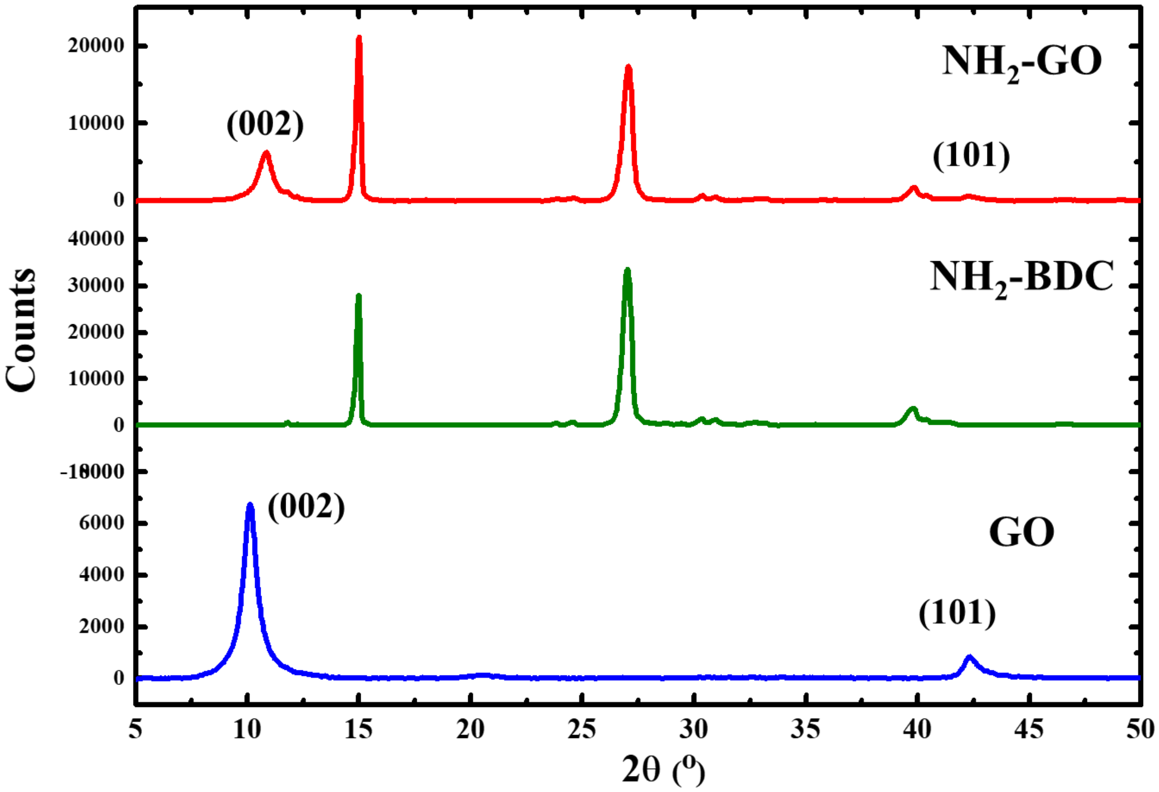

Figure S4. XRD analysis of graphene oxide (GO), amine BDC (NH_2-BDC), and amine functionalized graphene oxide (NH_2-GO). A sharp peak at around 10.8° and 42.5°, corresponding to the (002) and (101) plane, respectively, was observed in the GO, in compliance with previous reports [10]. These peaks were also observed in the NH_2-GO along with some other peaks, contributed to the presence of amino-terephthalic acid. The interlayer spacing (d) was calculated using Bragg's Law ($d= \lambda/(2\sin\theta)$), where d is the inter-layer spacing, λ is the wavelength of the X-ray (1.54060 Å) and θ is the diffraction angle) [11]. The interlayer spacing for GO and NH_2-GO was found to be 8.753 Å and 8.185 Å, respectively. These interlayer spacing values for GO are in line with the previous reports [12]. The decrease in the values of interlayer spacing can be due to the decomposition of some groups from the graphene oxide which leads to a more ordered graphene structure [13]. Using molecular modeling, Cunha et.al. showed that at some concentrations of functionalization, the interlayer spacing decreases. It is because to accommodate the functional groups, the graphene sheets flex, thereby leading to a decrease in the interlayer spacing value [14]. The crystallite size (calculated using Debye-Scherrer equation) [15] of GO and NH_2-GO is calculated to be 126.04 Å and 111.45 Å, respectively.

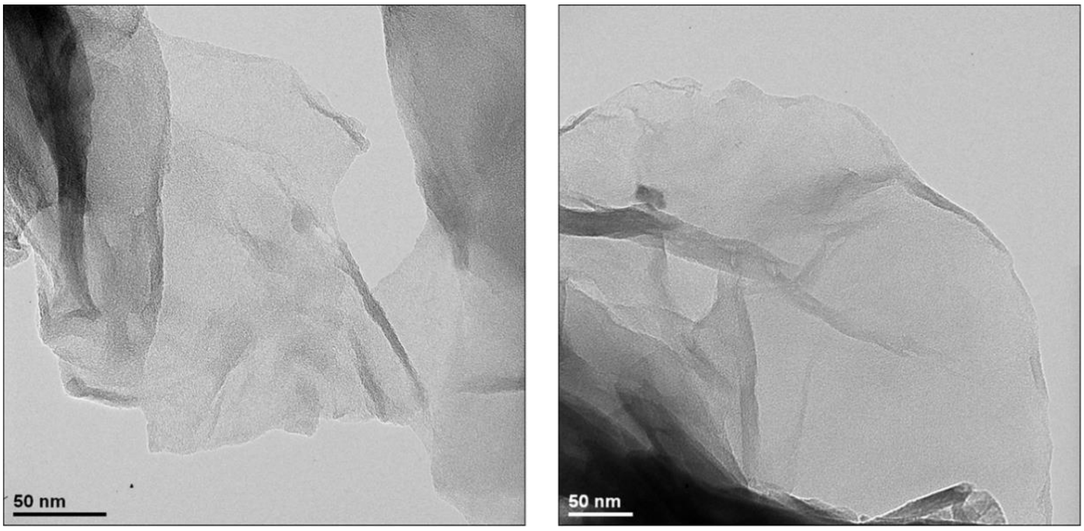

Figure S5. Two TEM images of NH_2-GO showing presence of thin sheets. 1 mg·mL^{-1} of NH_2-GO prepared in deionized water was dispersed in Milli Q water and sonicated for 30 min prior to drop casting onto the lacy carbon TEM grid. The suspension of the sample was drop casted on the grid and was allowed to air dry. Graphene oxide has a characteristic sheet like structure which is also evident from the current TEM images [16]. This image shows that the material has the appearance of thin sheets which matches with the reported nature of graphene oxide. The TEM image of the research reported done by Caliman et al. also showed that the amine functionalized graphene oxide material shows a lesser degree of aggregation as compared to the unfunctionalized graphene oxide [17].

2. Effect of scan rate

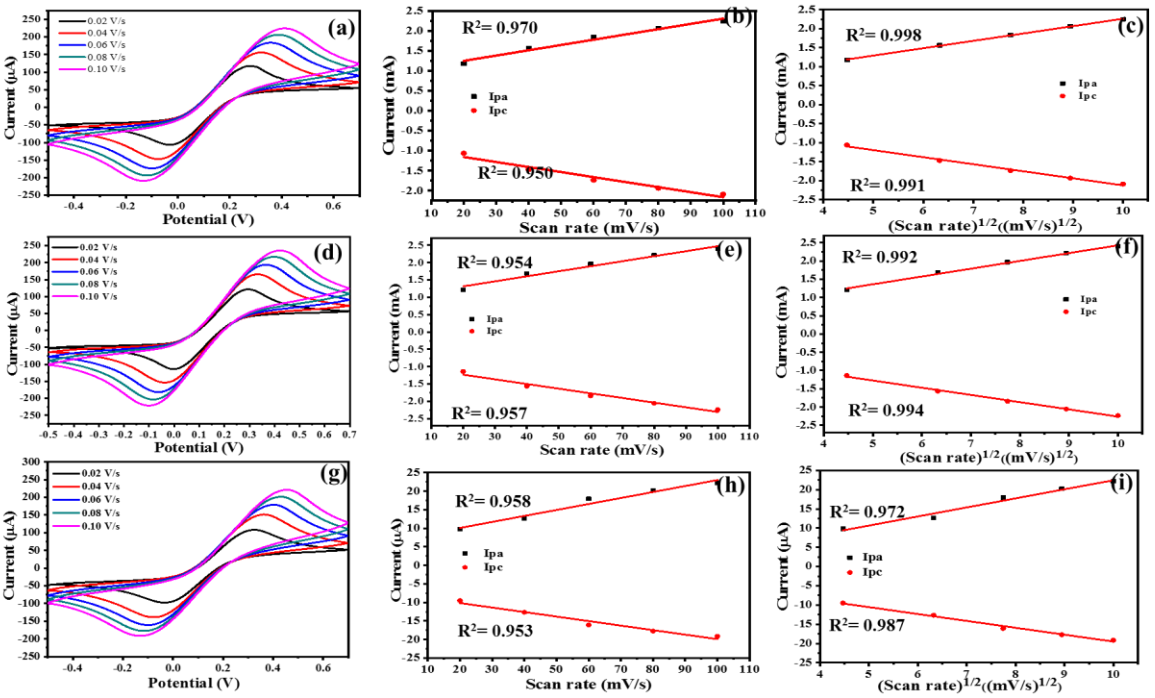

Figure S6. (a-c) Effect of scan rate for bare SPCE showing (a) cyclic voltammograms, (b) a plot of current versus scan rate and (c) a plot of current versus square root of scan rate. (d-f) Effect of scan rate for NH_2-GO@SPCE showing (d) cyclic voltammograms, (e) a plot of current versus scan rate and (f) a plot of current versus square root of scan rate. (g-i) Effect of scan rate for Ab/NH_2-GO@SPCE showing (g) cyclic voltammograms, (h) a plot of current versus scan rate and (i) a plot of current versus square root of scan rate.

3. Effect of Flow Rate

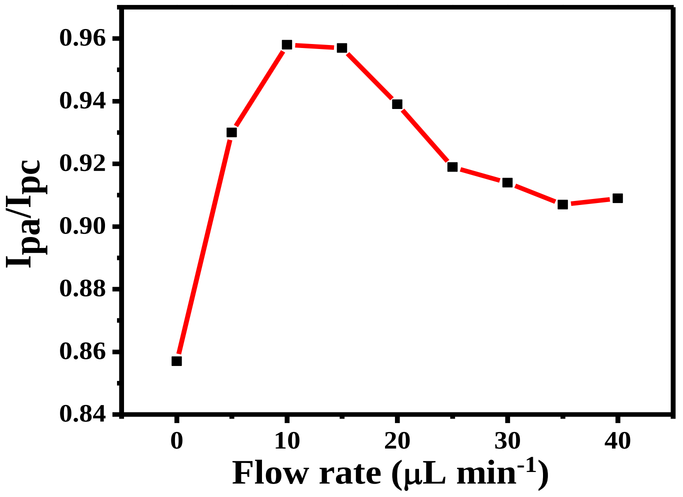

Figure S7. Figure shows the trend of ratio of the anodic to the cathodic current (I_{pa}/I_{pc}) at different flow rates.

4. Calibration Curve from Commercial ELISA Kit

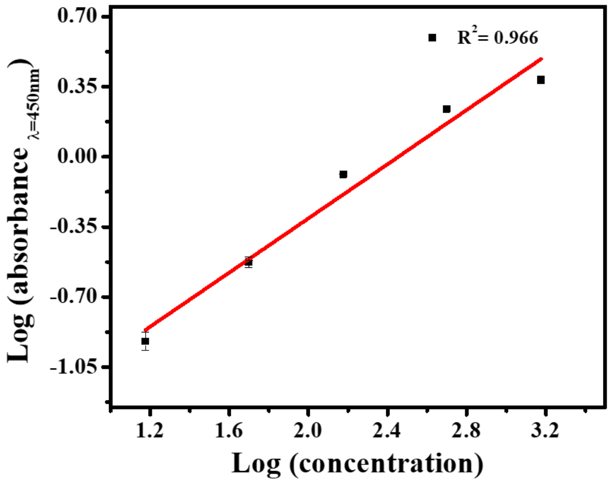

Figure S8. Linear response of ELISA in the concentration range of 0-1500 ng·mL^{-1} with an R^2 value of 0.966.

References

1. Hummers, W.S.; Offeman, R.E. Preparation of Graphitic Oxide. *J. Am. Chem. Soc.* **1958**, *80*, 1339–1339.
2. Chakraborty, A.K.; Saha, S.; Biswas, K.; Barbezat, M.; Dhalnak, V.R.; Terrasi, G. High yield synthesis of amine functionalized graphene oxide and its surface properties. *RSC Adv.* **2016**, *6*, 67916–67924.
3. Gupta, A.; Bhardwaj, S.K.; Sharma, A.L.; Deep, A. A graphene electrode functionalized with aminoterephthalic acid for impedimetric immunosensing of Escherichia coli. *Microchim. Acta* **2019**, *186*, 800.
4. Wazir, A.; Kundi, I.W. Synthesis of graphene nano sheets by the rapid reduction of electrochemically exfoliated graphene oxide induced by microwaves. *J. Chem. Soc. Pak.* **2016**, *38*, 11–16.
5. Karabacak, M.; Cinar, M.; Unal, Z.; Kurt, M.; Çınar, M. FT-IR, UV spectroscopic and DFT quantum chemical study on the molecular conformation, vibrational and electronic transitions of 2-aminoterephthalic acid. *J. Mol. Struct.* **2010**, *982*, 22–27.
6. Emiru, T.F.; Ayele, D.W. Controlled synthesis, characterization and reduction of graphene oxide: A convenient method for large scale production. *Egypt. J. Basic Appl. Sci.* **2017**, *4*, 74–79.
7. Ravikumar, L.; Saravanan, R.; Saravanamani, K.; Karunakaran, M. Synthesis and Characterization of New Polyamides with Substitutions in the Pendent Benzylidene Rings. *Des. Monomers Polym.* **2009**, *12*, 291–303.
8. Fang, S.; Huang, D.; Lv, R.; Bai, Y.; Huang, Z.-H.; Gu, J.; Kang, F. Three-dimensional reduced graphene oxide powder for efficient microwave absorption in the S-band (2–4 GHz). *RSC Adv.* **2017**, *7*, 25773–25779.
9. Wang, H.; Xie, J.; Almkhelfe, H.; Zane, V.; Ebini, R.; Sorensen, C.M.; Amama, P.B. Microgel-assisted assembly of hierarchical porous reduced graphene oxide for high-performance lithium-ion battery anodes. *J. Mater. Chem. A* **2017**, *5*, 23228–23237.
10. Nechiyil, D.; Vinayan, B.P.; Ramaprabhu, S. Tri-iodide reduction activity of ultra-small size PtFe nanoparticles supported nitrogen-doped graphene as counter electrode for dye-sensitized solar cell. *J. Colloid Interface Sci.* **2017**, *488*, 309–316.
11. Bragg, W.H.; Bragg, W.L. The reflection of X-rays by crystals. Proceedings of the Royal Society of London. Series A, Containing Papers of a Mathematical and Physical Character. *R. Soc.* **1913**, *88*, 428–438.
12. Sharma, R.; Chadha, N.; Saini, P. Determination of defect density, crystallite size and number of graphene layers in graphene analogues using X-ray diffraction and Raman spectroscopy. *Indian J. Pure Appl. Phys.* **2017**, *55*, 625–629.
13. Huang, Y.; Gong, Q.; Zhang, Q.; Shao, Y.; Wang, J.; Jiang, Y.; Zhao, M.; Zhuang, D.; Liang, J. Fabrication and molecular dynamics analyses of highly thermal conductive reduced graphene oxide films at ultra-high temperatures. *Nanoscale* **2017**, *9*, 2340–2347.
14. Cunha, E.; Proença, F.; Costa, F.M.; Fernandes, A.J.; Ferro, M.; Lopes, P.E.; Debs, M.G.; Melle-Franco, M.; Deepak, F.L.; Paiva, M.C. Self-assembled functionalized graphene nanoribbons from carbon nanotubes. *ChemistryOpen* **2015**, *4*, 115–119.
15. Patterson, A.L. The Scherrer Formula for X-Ray Particle Size Determination. *Phys. Rev.* **1939**, *56*, 978–982.
16. Inagaki, M.; Kang, F. *Chapter 3-Engineering and Applications of Carbon Materials, in Materials Science and Engineering of Carbon: Fundamentals (Second Edition)*; Inagaki, M., Kang, F., Eds.; Butterworth-Heinemann: Oxford, UK, 2014; pp. 219–525.
17. Caliman, C.C.; Mesquita, A.F.; Cipriano, D.F.; Freitas, J.C.C.; Cotta, A.A.; Macedo, W.A.A.; Porto, A. One-pot synthesis of amine-functionalized graphene oxide by microwave-assisted reactions: An outstanding alternative for supporting materials in supercapacitors. *RSC Adv.* **2018**, *8*, 6136–6145.
